# Association between serum 25(OH)D and risk of all-cause mortality in adults with prior cardiovascular disease: a cohort study from NHANES 2007–2018

**DOI:** 10.1186/s12872-023-03257-0

**Published:** 2023-05-06

**Authors:** Ben Hu, Jian Chen, Yihang Shi, Linlin Hou

**Affiliations:** 1 Department of Cardiology, The Second People’s Hospital of Hefei, Hefei Hospital Affiliated to Anhui Medical University, Hefei, 230011 Anhui China; 2 Anhui Medical University, Hefei, 230000 Anhui China

**Keywords:** Vitamin D, Cohort study, Mortality, Cardiovascular disease, NHANES

## Abstract

**Background:**

Serum vitamin D deficiency is common in the patients with cardiovascular disease (CVD), but the association between serum vitamin D levels and risk of all-cause mortality in patients with CVD is controversial.

**Objective:**

This study aimed to understand better the association between serum 25(OH)D status and risk of all-cause mortality in patients with prior CVD.

**Method:**

We conducted a cohort study using data from the National Health and Nutrition Examination Survey from 2007 to 2018 to investigate the association between serum 25(OH)D and the risk of all-cause mortality using multivariate Cox regression models, with further subgroup analyses and interactions smooth curve fitting to address possible nonlinearities.

**Result:**

A total of 3220 participants with prior CVD were included in this study, with a total of 930 deaths over a median follow-up of 5.52 years, with multivariable-adjusted serum vitamin D levels after natural log transformation (4.31–4.5 ) as a reference in COX regression, and corrected HRs and 95% CIs of 1.81 (1.31, 2.50), 1.34 (1.07, 1.66), 1.28 (1.05, 1.56),1.00 (reference), 1.10 (0.89, 1.37) for all-cause mortality, respectively. Results remained robust in the stratified analysis of interactions, but a L-shaped relationship was detected. We identified an inflection point of 4.5 after multivariate adjustment through a two-stage linear regression model and recursive algorithm.

**Conclusion:**

Our findings demonstrate that increasing serum 25(OH)D levels may have a L-shaped relationship with risk of all-cause mortality and that increases in serum 25(OH)D levels do not continue to reduce the risk of all-cause mortality.

**Supplementary Information:**

The online version contains supplementary material available at 10.1186/s12872-023-03257-0.

## Introduction

Cardiovascular disease is the leading cause of death worldwide [1]. It is also the leading cause of death in the United States (US), posing a significant economic and social burden, and the incidence of cardiovascular disease (CVD) continues to increase in the US population, increasingly affecting younger people [2].

Vitamin D is a hormone that primarily regulates calcium and phosphate metabolism [3]. It plays a role in the function of endothelial cells and glucose homeostasis, inhibiting PTH and regulating the renin-angiotensin-aldosterone system (RAAS) [4–8]. And it may attenuate oxidative stress, inflammatory and immune responses, thrombosis, and calcium and lipoprotein regulation [5]. In several large prospective studies and meta-analyses, vitamin D deficiency was considered an independent risk factor for cardiovascular outcomes and all-cause mortality [9–12]. Meanwhile, there is also an inverse relationship between serum 25-hydroxyvitamin D (25OHD) levels and CVD mortality [13, 14]. In addition, several prospective observational studies have reported that high serum vitamin D levels may be detrimental to CVD or all-cause mortality [15, 16]. Despite the accumulating mechanistic and population evidence for vitamin D prevention of CVD, published randomized controlled trials have not detected any effect of vitamin D supplementation on sudden CVD [17, 18]. They have failed to confirm the cardiovascular benefits of vitamin D supplementation in the general population or those without CVD [19, 20]. Vitamin D status remains a significant global public health issue [21]. In addition, past studies have limitations, such as small sample sizes, neglecting confounding factors such as family income, exercise status, presence of other diseases and vitamin supplementation, or inadequate adjustment for some significant covariates, which may have influenced the results. Therefore, we sought to understand better the association between vitamin D status and risk of mortality in patients with prior CVD. This study utilized the 2007–2018 National Health and Nutrition Examination Survey (NHANES) database to assess vitamin D supplementation’s potential benefits in prior CVD patients.

## Materials and methods

### Data source and study population

**Fig. 1:**
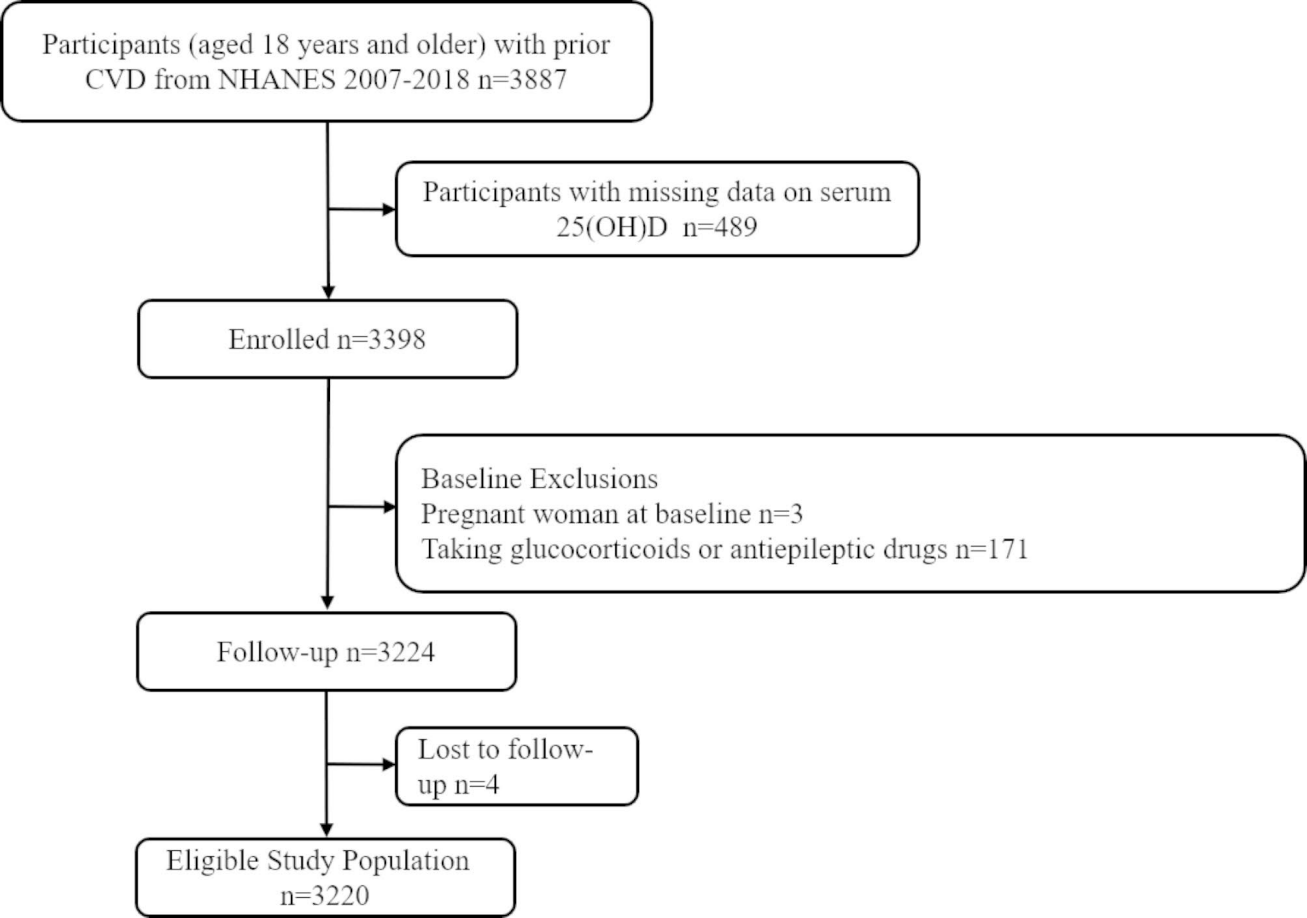
Flow diagram of study

The National Health and Nutrition Examination Survey (NHANES) is a large, nationally representative statistical survey. It was designed to understand the health and nutrition status among the general U.S. population, expand national health knowledge, and address emerging public health issues. This study used NHANES data from the last 12 years (2007–2018) and was limited to patients aged 18 years or older with prior CVD. Among the 3887 eligible patients, 489 patients with incomplete serum 25(OH)D data, 171 patients taking glucocorticoids or antiepileptic drugs that could affect serum 25(OH)D levels, three pregnant patients, and four participants who were lost to follow-up were excluded. Ultimately, 3220 participants were included in the analysis (Fig. 1). All study protocols of NHANES were approved by the Institutional Review Board of the National Center for Health Statistics (NCHS). All participants provided written informed consent.

### Study variables

The primary variable was serum 25(OH)D levels. Serum samples were examined at the National Center for Environmental Health, the Centers for Disease Control and Prevention, and the Laboratory Sciences Division in Atlanta, GA. High-performance liquid chromatography tandem mass spectrometry (HPLC-MS/MS) was used to quantify 25-hydroxyvitamin D3 (25OHD3), 3-epi-25-hydroxyvitamin D3 (epi-25OHD3) and-25-hydroxyvitamin D2 (25OHD2) in human serum samples. Serum 25(OH)D concentration was equal to 25-hydroxyvitamin D2 (25OHD2) concentration plus 25-hydroxyvitamin D3 (25OHD3) concentration.

Regarding the question, “Have you ever been diagnosed with coronary heart disease, congestive heart failure, angina pectoris, heart attack, or stroke by a physician or other health professional?”, Those who responded “yes” were considered to have prior CVD.

Potential confounding variables were included in the analysis. Sociodemographic factors included age, sex (male, female), race/ethnicity (Non-Hispanic White, Non-Hispanic Black, Mexican American, and others), education level (below high school, high school, above high school), smoking status (never smoker, former smoker, current smoker), drinking status ( nondrinker, low-to-moderate drinker, heavy drinker), family income-poverty ratio (≤ 1.0, 1–3, > 3), leisure-time physical activity (inactive, insufficiently active, active), heart rate, BMI, and health-related illnesses including diabetes (yes), hypertension (yes), hyperlipidemia (yes), kidney failure (yes), cancer (yes), and, family history of heart disease (yes). Laboratory tests included hemoglobin, white blood cells, lymphocytes, monocytes, triglycerides, total cholesterol, high-density lipoprotein (HDL), low-density lipoprotein (LDL), fasting blood glucose, serum creatinine, serum uric acid, aspartate aminotransferase (AST), alanine aminotransferase (ALT), albumin, glycated hemoglobin, calcium, and phosphorus. In addition, for smoking status, participants who reported smoking less than 100 cigarettes in their lifetime were considered as never smokers. Participants who had smoked more than 100 cigarettes throughout their lifetime but had quit smoking were classified as former smokers, whereas those who were still smoking were classed as current smokers. Drinking status was categorized as a nondrinker, low-to-moderate drinker (< 2 drinks/day in men and < 1 drink/day in women), or heavy drinker (≥ 2 drinks/day in men and ≥ 1 drink/day in women). Physical activity levels were assessed using metabolic equivalent (MET) intensity levels [22]. Leisure-time physical activity included inactive (metabolic equivalent < 3), insufficiently active (metabolic equivalent3-6), and active (metabolic equivalent > 6) [23]. MET scores were calculated according to Additional file 1 [24].

### Outcomes

The primary outcome of our study was risk of all-cause mortality. The time of the incident was calculated from the collection of the blood sample to the date of death or census (December 31, 2019). All studies were conducted according to the guidelines, and detailed information about NHANES is available at https://www.cdc.gov/nchs/nhanes/. Publicly available.

### Statistical analysis

Vitamin D grading was based on serum 25(OH)D levels. The Endocrine Society Clinical Practice Guidelines consider severe vitamin D deficiency when serum 25 (OH) D levels are below 25.0 nmol/L, moderate deficiency when serum 25 (OH) D levels are between 25.0 and 49.9 nmol/L, insufficient when serum 25 (OH) D levels are between 50.0 and 74.9 nmol/L, and sufficient when serum 25 (OH) D levels are higher than 75 nmol/L [25]. Mean ± standard deviation (SD) or median (interquartile range) for continuous variables and frequency (percentage) for categorical variables according to baseline serum 25(OH)D categories. Chi-squared test (for categorical variables), One-Way ANOVA test (for normal distribution), and Kruskal-Wallis H-test (for skewed distribution) were used to test for differences among different serum 25(OH)D.

Serum 25(OH)D concentration was not normally distributed, thus it was natural log-transformed. Hazard ratios (HRs) and 95% confidence intervals (CIs) were calculated for associations between serum 25(OH)D concentration after natural log transformation and all-cause mortality using univariate and multivariable Cox regression models. The reference group consisted of individuals with serum 25(OH)D after natural log transformation (4.31–4.50). Minimally-adjusted model adjusted for age, sex, race/ethnicity, education level, and family income-poverty ratio. Fully-adjusted model successively adjusted for age, sex, race/ethnicity, education level, smoking status, renal failure, hypercholesterolemia, diabetes, cancer, family history of heart disease, family income-poverty ratio, BMI, WBC, HDL, TC glucose, albumin, creatinine, leisure-time physical activity, calcium, and glycated hemoglobin. Additionally, stratified analyses were conducted based on gender, age, race, education level, smoking status, hypercholesterolemia, renal failure, family income-poverty ratio, leisure-time physical activity, glycohemoglobin, and BMI. The adjustment strategy is the same as in the fully-adjusted model except the variable itself. Log-likelihood ratio test was used to assess the interaction effects between serum 25(OH)D concentration and subgroup variables. No adjustments were made for multiple comparisons.

**Fig. 2:**
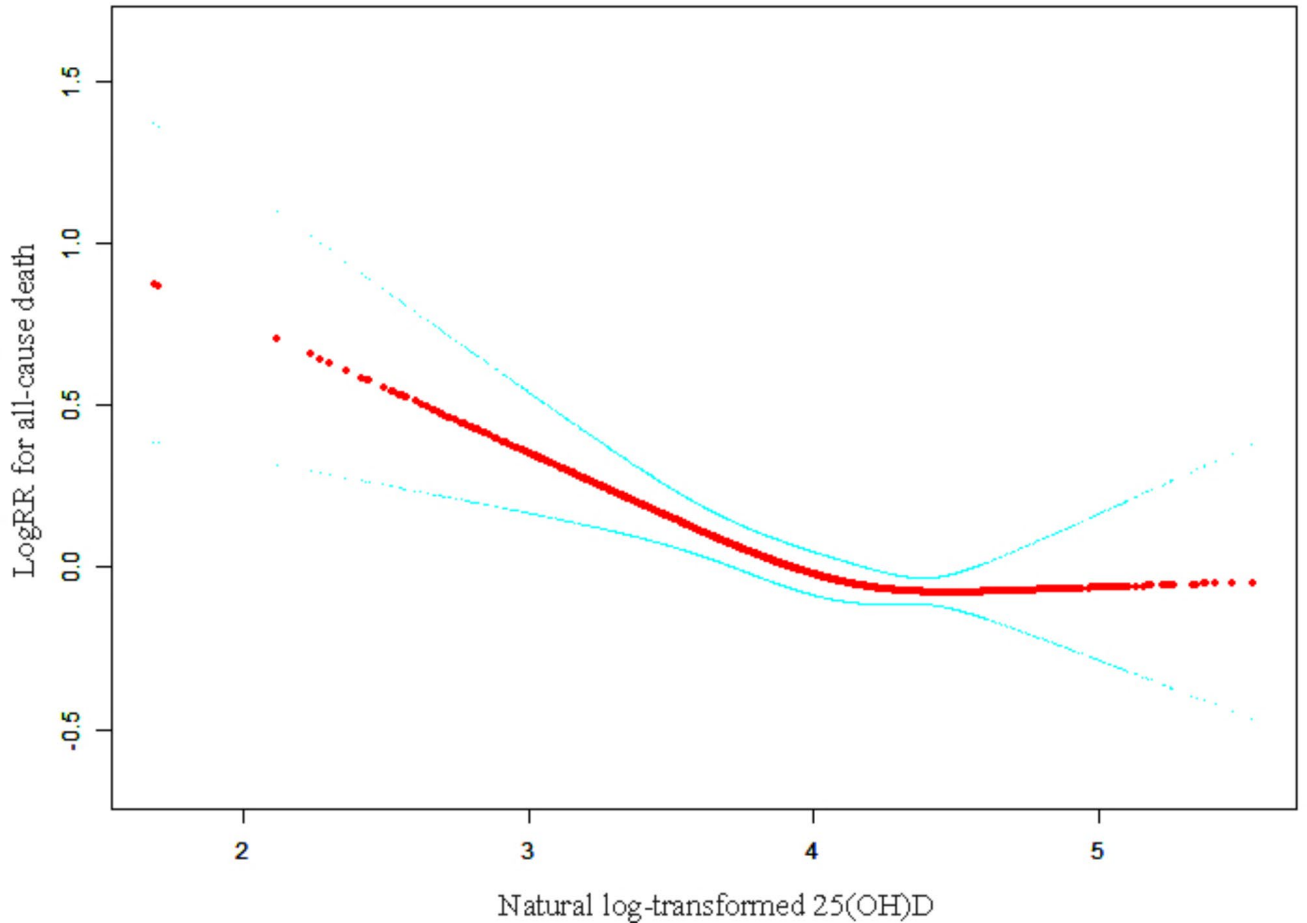
Smooth curve fitting demonstrates the relationship between serum 25(OH)D after natural log transformation and risk of all-cause death. The predicted log(relative risk) for all-cause death in the y-axis and the continuous covariate in the x-axis. Smooth curve fit is indicated by the solid red line. The 95% confidence interval is represented by the blue band. The adjustment strategy is the same as the fully-adjusted model

We further applied a two-piecewise linear regression model to examine the threshold effect of serum 25(OH)D on logHR using a smooth function (Fig. 2). Threshold levels (i.e., turning points) were determined using trial and error, choosing the turning points that give the greatest model possibilities. A single-line linear regression model and a two-stage linear regression model were also investigated for their log-likelihood ratios. In order to avoid the reduction of statistical test efficiency and bias caused by direct exclusion of missing values, we used multiple imputation, based on 5 replications and a chained equation approach method in the R MI procedure, to account for missing data. The newly generated five sets of data were used for Cox regression analysis and their results were combined. All analyses were conducted using Empower (R) (www.empowerstats.com) and R (http://www.R-project.org).

## Results

A total of 3220 patients with CVD (aged 66.3 ± 12.7 years and 1837 [57%] men) were included in this analysis. Table 1 shows the baseline characteristics according to serum 25(OH)D levels. Among them, 159 (5%) had severe vitamin D deficiency, 769 (23.8%) had a moderate deficiency, 1017 (31.6%) had inadequacy, and 1275 (39.6%) were adequate. Compared to patients with severe vitamin D deficiency, patients with higher serum 25 (OH)D concentrations were more likely to be elderly, male, accept higher education, current non-smokers, with higher family income, less drink, and more exercise. They had lower BMI, slower heart rate, higher prevalence of hyperlipidemia, lower prevalence of diabetes, lower percentage of glycosylated hemoglobin, but higher prevalence of cancer, higher white blood cells, lower LDL, lower total cholesterol, higher serum creatinine, higher albumin, and higher survival ratio.

**Table 1:** Baseline characteristics of participants

	< 25 nmol/L	Serum 25(OH)D concentrations, nmol/L			P-value
		25-49.9 nmol/L	50-74.9 nmol/L	≥ 75 nmol/L	
N	159	769	1017	1275	
Age (mean ± SD), years	60.93 ± 13.47	62.61 ± 13.49	65.41 ± 12.69	69.84 ± 11.02	< 0.001
Sex n (%)					< 0.001
Male	69 (43.40%)	431 (56.05%)	632 (62.14%)	705 (55.29%)	
Female	90 (56.60%)	338 (43.95%)	385 (37.86%)	570 (44.71%)	
Race/ethnicity n (%)					< 0.001
Mexican American n (%)	11 (6.92%)	111 (14.44%)	121 (11.90%)	70 (5.49%)	
Non-Hispanic Black	90 (56.60%)	271 (35.24%)	169 (16.62%)	176 (13.80%)	
Non-Hispanic White	42 (26.42%)	268 (34.85%)	521 (51.22%)	862 (67.61%)	
Other	16 (10.06%)	119 (15.47%)	206 (20.26%)	167 (13.10%)	
Education level n (%)					< 0.001
Below high school	63 (39.62%)	294 (38.23%)	364 (35.79%)	349 (27.37%)	
High school	39 (24.53%)	194 (25.23%)	264 (25.96%)	308 (24.16%)	
Above high school	57 (35.85%)	281 (36.54%)	389 (38.25%)	618 (48.47%)	
Smoking status n (%)					< 0.001
Never smoker	64 (40.25%)	284 (36.93%)	389 (38.25%)	550 (43.17%)	
Former smoker	40 (25.16%)	258 (33.55%)	412 (40.51%)	532 (41.76%)	
Current smoker	55 (34.59%)	227 (29.52%)	216 (21.24%)	192 (15.07%)	
Drinking status n (%)					0.964
Nondrinker	39 (27.08%)	198 (28.41%)	271 (28.65%)	347 (28.94%)	
Low-to-moderate drinker	95 (65.97%)	454 (65.14%)	624 (65.96%)	777 (64.80%)	
Heavy drinker	10 (6.94%)	45 (6.46%)	51 (5.39%)	75 (6.26%)	
Renal failure n (%)	15 (9.43%)	84 (10.92%)	90 (8.85%)	174 (13.65%)	0.003
Hypertension n (%)	128 (80.50%)	577 (75.03%)	729 (71.68%)	969 (76.00%)	0.031
Hypercholesterolemia n (%)	75 (47.17%)	445 (57.87%)	630 (61.95%)	809 (63.45%)	< 0.001
Diabetes n (%)	64 (40.25%)	298 (38.75%)	343 (33.73%)	416 (32.63%)	0.014
Cancer n (%)	20 (12.58%)	125 (16.25%)	188 (18.49%)	339 (26.59%)	< 0.001
Family history of heart disease n (%)	37 (23.27%)	170 (22.11%)	223 (21.93%)	281 (22.04%)	0.985
Family income-poverty ratio n (%)					< 0.001
≤ 1.0	50 (35.21%)	235 (33.33%)	237 (25.73%)	211 (18.08%)	
1–3	70 (49.30%)	336 (47.66%)	458 (49.73%)	567 (48.59%)	
> 3	22 (15.49%)	134 (19.01%)	226 (24.54%)	389 (33.33%)	
Leisure-time physical activity n (%)					< 0.001
Inactive	86 (54.09%)	382 (49.67%)	424 (41.69%)	535 (41.96%)	
Insufficiently active	39 (24.53%)	198 (25.75%)	260 (25.57%)	343 (26.90%)	
Active	34 (21.38%)	189 (24.58%)	333 (32.74%)	397 (31.14%)	
Status n (%)					0.038
Survive	99 (62.26%)	537 (69.83%)	726 (71.39%)	928 (72.78%)	
Death	60 (37.74%)	232 (30.17%)	291 (28.61%)	347 (27.22%)	
N	159	769	1017	1275	
Heart rate (mean ± SD), bpm	76.36 (12.40)	72.31 (12.92)	70.06 (12.18)	68.65 (11.37)	< 0.001
BMI kg/m2	33.86 (10.24)	31.62 (7.76)	30.77 (7.04)	29.34 (6.64)	< 0.001
Hemoglobin (g/dL)	13.13 (1.87)	13.71 (1.71)	13.97 (1.63)	13.72 (1.55)	< 0.001
WBC (1000 cells/uL)	7.22 (2.45)	7.43 (2.31)	7.56 (2.82)	7.51 (4.66))	0.044
NEUT (1000 cells/uL)	4.26 (1.82)	4.51 (1.83)	4.52 (1.67)	4.43 (1.66)	0.219
LYM (1000 cells/uL)	2.13 (0.93)	2.05 (0.75)	2.13 (1.80)	2.17 (4.12)	< 0.001
HDL (mg/dL)	51.68 (21.04)	48.52 (15.37)	47.92 (14.77)	51.85 (16.18)	< 0.001
Triglyceride(mg/dL)	123.00 (69.50–199.00)	135.00 (78.00-209.00)	130.00 (82.00-212.00)	124.00 (77.00-204.00)	0.100
LDL (mg/dL)	103.68 (41.38)	101.58 (37.18)	99.95 (37.97)	96.55 (34.73)	0.006
TC (mg/dL)	182.54 (47.25)	180.13 (46.43)	178.49 (46.19)	176.44 (42.17)	0.179
Glucose (mg/dL)	101.00 (91.00-127.00)	102.00 (91.00-129.50)	102.00 (92.00-126.00)	100.00 (90.00-120.00)	< 0.001
Creatinine (µmol/L)	83.98 (66.30-103.43)	84.86 (69.84-104.75)	85.75 (71.38–105.20)	90.17 (75.14-113.15)	0.014
Serum uric acid (mg/dL)	6.33 (1.80)	5.95 (1.65)	5.99 (1.57)	6.00 (1.63)	0.057
AST (U/L)	21.00 (17.00–26.00)	22.00 (18.00–27.00)	23.00 (19.50–28.00)	23.00 (19.00–28.00)	< 0.001
ALT (U/L)	17.00 (13.00–24.00)	19.00 (14.00–26.00)	21.00 (16.00–27.00)	19.00 (15.00–25.00)	< 0.001
Albumin (g/dL)	3.90 (0.49)	4.03 (0.36))	4.13 (0.32)	4.15 (0.33)	< 0.001
Phosphorus (mmol/L)	1.19 (0.21)	1.20 (0.20)	1.18 (0.18)	1.20 (0.19)	0.168
Calcium (mmol/L)	2.32 (0.13)	2.32 (0.10)	2.34 (0.09)	2.36 (0.10)	< 0.001
Glycohemoglobin (%)	6.47 (1.67)	6.51 (1.63)	6.29 (1.28)	6.16 (1.11)	< 0.001

Mean ± SD or median (interquartile range) for continuous variables and as frequencies (percentages) for categorical variables

Abbreviations: BMI: body mass index; WBC: white blood cell; NEUT: neutrophils; LYM: lymphocyte; TC: total cholesterol; HDL: high-density lipoprotein; LDL: low-density lipoprotein; AST: aspartate aminotransferase; ALT: alanine aminotransferase

All sociodemographic and health-related disease factors were statistically significant at different serum 25(OH)D classification levels except drinking status at baseline, and family history of heart disease. In laboratory tests, monocytes, total cholesterol, triglycerides, serum uric acid and serum phosphate were not statistically significant with different serum 25(OH)D subgroup levels.

We used smooth curve fitting to intuitively evaluate the functional relationship between serum 25(OH)D after natural log-transformed and the risk of all-cause death (Fig. 2). In the Fig. 2, a log (relative risk) of 0 means that the relative risk is 1 (having no effect on all-cause death), while a log (relative risk) of 1 means that the relative risk is 2.71 (i.e., the probability of all-cause death increases by 2.71 times). Using a two-stage linear regression model and a recursive algorithm and adjusting for variables, the inflection point was estimated to be 4.5 (Table 2).

**Table 2:** Nonlinearity addressing of serum 25(OH)D level after natural log transformation and all-cause mortality

Outcome:	HR,95%CI, P value
Fitting model by standard linear regression	0.75 (0.65, 0.87) 0.0109
Fitting model by two-piecewise linear regression	
Inflection point	4.5
< 4.5	0.72 (0.61, 0.85) < 0.0001
> 4.5	1.16 (0.52, 2.61) 0.7114
P for the log-likelihood ratio test	0.285

The adjustment strategy is the same as the fully-adjusted model

During 18,560 person years of follow-up, 930 deaths (median 5.25 years), three multivariable Cox regression models were used to explore the association between serum 25(OH)D after natural log-transformed and risk of all-cause mortality (Table 3). In the Cox regression analysis, patients with severe vitamin D deficiency, moderate deficiency, and insufficiency had a higher risk of all-cause mortality than patients with serum 25(OH)D after natural log-transformed of 4.31–4.5 (Table 3). In the fully adjusted model, hazard ratios (HRs) and 95% confidence intervals (CIs) from lowest to highest serum 25(OH)D categories after natural log-transformed (< 3.22, 3.22–3.91, 3.91–4.31, 4.31–4.5, and ≥ 4.5) were 1.81 (1.31, 2.50), 1.34 (1.07, 1.66), 1.28 (1.05, 1.56), 1.00 (reference), and 1.10 (0.89, 1.37).

**Table 3:** Univariate and multivariate results by cox regression

Exposure	Non-adjusted model HR, 95%CI, P	Minimally-adjusted model HR, 95%CI, P	Fully-adjusted model HR, 95%CI, P
Serum 25(OH)D concentrations (natural log transformation)			
Q1 (< 3.22)	1.39 (1.03, 1.88) 0.0302	2.23 (1.63, 3.05) < 0.0001	1.81 (1.31, 2.50) 0.0003
Q2(≥ 3.22, < 3.91)	1.06 (0.87, 1.31) 0.5530	1.51 (1.22, 1.87) 0.0002	1.34 (1.07, 1.66) 0.0091
Q3(≥ 3.91, < 4.31)	1.02 (0.84, 1.24) 0.8207	1.26 (1.03, 1.53) 0.0233	1.28 (1.05, 1.56) 0.0157
Q4(≥ 4.31, < 4.5)	Ref	Ref	Ref
Q5(≥ 4.5)	1.23 (1.00, 1.52) 0.0535	1.16 (0.94, 1.44) 0.1638	1.10 (0.89, 1.37) 0.3826
P for trend	0.2543	0.0014	0.0113

Non-adjusted model: no covariates were adjusted. Minimally-adjusted model: we only adjusted for age, sex; race/ethnicity; education level; family income-poverty ratio. Fully-adjusted model: we adjusted for age, sex, race/ethnicity, education level, smoking status, renal failure, diabetes, hypercholesterolemia, cancer, family history of heart disease, family income-poverty ratio, leisure-time physical activity, BMI, WBC, HDL, TC, Glu, Albumin, Creatinine, Calcium, and Glycohemoglobin

After adjusting the variables, a stratified analysis of the relationship between serum 25(OH)D after natural log-transformed and the risk all-cause mortality is shown in Table 4. The population with serum 25(OH)D after natural log-transformed of 4.31-4.5was used as reference. When analyses were stratified by gender, age, race, education level, smoking status, hypercholesterolemia, renal failure, family income-poverty ratio, leisure-time physical activity, glycohemoglobin, and BMI, consistent results were observed in people over age 60 who were male, white people ,high school educated, former smoker, and had BMI(25–30), family income-poverty ratio(1–3), glycohemoglobin(< 7%) and played active physical activity. We tested the robustness of the association between serum 25(OH)D and the risk all-cause mortality in different subgroups by interaction. In the model, the interaction tests for covariates with serum 25(OH)D were non-significant except for BMI.

**Table 4:** Subgroup analyses by the stratified cox regression model

	N	Q1(< 3.22)	Serum 25(OH)D concentrations (natural log transformation)			Q5(≥ 4.5)	P for interaction
			Q2(≥ 3.22, < 3.91)	Q3(≥ 3.91, < 4.31)	Q4(≥ 4.31, < 4.5)		
Age, years							0.2479
< 60	811	2.99 (1.17, 7.66)	1.28 (0.56, 2.94)	0.91 (0.39, 2.12)	Ref	0.70 (0.22, 2.22)	
≥ 60	2409	1.55 (1.08, 2.21)	1.25 (0.99, 1.57)	1.24 (1.01, 1.53)	Ref	1.11 (0.89, 1.39)	
Sex							0.7969
Male	1837	1.79 (1.11, 2.88)	1.42 (1.07, 1.88)	1.31 (1.02, 1.69)	Ref	1.08 (0.82, 1.44)	
Female	1383	1.79 (1.14, 2.81)	1.11 (0.77, 1.60)	1.14 (0.81, 1.59)	Ref	1.05 (0.74, 1.49)	
Race							0.27913
White	1693	1.63 (0.99, 2.69)	1.43 (1.08, 1.89)	1.41 (1.11, 1.79)	Ref	1.27 (0.99, 1.63)	
Non-White	1527	1.48 (0.94, 2.35)	1.05 (0.73, 1.51)	0.96 (0.66, 1.41)	Ref	0.72 (0.45, 1.16)	
Education level							0.0829
below high school	1070	1.72 (1.06, 2.80)	1.09 (0.78, 1.53)	1.20 (0.88, 1.64)	Ref	0.95 (0.65, 1.39)	
high school	805	2.87 (1.40, 5.88)	2.75 (1.66, 4.58)	1.91 (1.21, 3.04)	Ref	1.81 (1.11, 2.96)	
above high school	1345	1.75 (0.97, 3.15)	1.16 (0.80, 1.69)	1.00 (0.71, 1.41)	Ref	0.89 (0.63, 1.25)	
Smoking status							0.6829
never smoker	1287	1.92 (1.14, 3.23)	1.36 (0.94, 1.96)	1.37 (0.97, 1.92)	Ref	1.24 (0.87, 1.76)	
former smoker	1242	2.15 (1.26, 3.67)	1.58 (1.13, 2.19)	1.44 (1.06, 1.94)	Ref	1.14 (0.83, 1.56)	
current smoker	691	1.47 (0.73, 2.96)	0.88 (0.52, 1.48)	0.84 (0.50, 1.39)	Ref	0.67 (0.37, 1.22)	
Renal failure							0.5238
Yes	363	1.86 (0.71, 4.86)	1.58 (0.79, 3.19)	1.73 (0.89, 3.34)	Ref	1.01 (0.50, 2.03)	
Other	2857	1.82 (1.29, 2.56)	1.27 (1.01, 1.60)	1.19 (0.96, 1.48)	Ref	1.07 (0.85, 1.35)	
Hypercholesterolemia							0.0837
Yes	2009	2.32 (1.52, 3.56)	1.24 (0.93, 1.64)	1.36 (1.05, 1.76)	Ref	1.12 (0.85, 1.47)	
No	1211	1.18 (0.71, 1.98)	1.45 (1.02, 2.06)	1.17 (0.85, 1.63)	Ref	1.00 (0.70, 1.43)	
Family income-poverty ratio							0.2406
≤ 1.0	807	3.14 (1.63, 6.04)	1.35 (0.84, 2.18)	1.29 (0.80, 2.07)	Ref	1.22 (0.69, 2.16)	
1–3	1576	1.82 (1.17, 2.84)	1.49 (1.10, 2.01)	1.42 (1.08, 1.85)	Ref	1.11 (0.83, 1.49)	
> 3	837	(0.32, 1.78) 0.5160	(0.67, 1.81) 0.6892	(0.60, 1.41) 0.6966	Ref	(0.61, 1.44) 0.7655	
Leisure-time physical activity							0.0576
Inactive	1427	1.38 (0.93, 2.04)	1.22 (0.93, 1.59)	1.09 (0.84, 1.42)	Ref	0.93 (0.70, 1.24)	
Insufficient	840	2.30 (1.08, 4.93)	1.07 (0.68, 1.69)	1.03 (0.69, 1.54)	Ref	1.15 (0.75, 1.77)	
Active	953	5.65 (2.14, 14.91)	2.99 (1.51, 5.90)	2.71 (1.51, 4.84)	Ref	1.76 (0.95, 3.29)	
Glycohemoglobin (%)							0.2576
< 7	2619	2.03 (1.41, 2.91)	1.48 (1.15, 1.89)	1.35 (1.08, 1.70)	Ref	1.21 (0.95, 1.54)	
≥ 7	598	1.04 (0.50, 2.19)	0.93 (0.58, 1.49)	0.99 (0.63, 1.58)	Ref	0.67 (0.39, 1.16)	
BMI, kg/m2							0.0462
< 25	686	0.94 (0.46, 1.94)	1.09 (0.71, 1.66)	0.99 (0.69, 1.43)	Ref	0.72 (0.49, 1.07)	
≥ 25, < 30	1036	3.24 (1.71, 6.15)	1.93 (1.29, 2.88)	1.82 (1.26, 2.61)	Ref	1.38 (0.94, 2.02)	
≥ 30	1498	1.72 (1.08, 2.73)	1.18 (0.84, 1.68)	1.07 (0.77, 1.51)	Ref	1.31 (0.90, 1.90)	

The adjustment strategy is the same as the fully-adjusted model except the variable itself

## Discussion

In this study, we used data from the NHANES (2007–2018) database to explore whether there is an independent association between serum 25(OH)D levels and risk of all-cause mortality in people with prior cardiovascular disease (CVD). In this large US cohort study based on the NHANES database, we found a non-linear relationship (L-shaped curve) between serum 25(OH)D and risk of all-cause mortality in patients with prior cardiovascular disease. No association was maintained when the serum 25(OH)D after natural log-transformed was higher than 4.5, a negative association was found when the serum 25(OH)D after natural log-transformed was lower than 4.5. Only 29% of patients with prior CVD in the cohort had insufficient serum 25(OH)D, possibly because of vitamin D supplementation. Understanding the level of vitamin D in patients with prior CVD and optimizing its range may help to improve the subsequent health status of patients with prior CVD.

Previously, attention has focused on the general CVD-free population, and observational studies have examined the relationship between vitamin D and all-cause or cause-specific mortality, with varying results. In a recent randomized placebo-controlled trial of older Australians, vitamin D supplementation did not reduce all-cause mortality [26]. In a New Zealand randomized controlled trial of community-dwelling adults aged 50 to 84 years (n = 5108) with a median follow-up of 3.3 years, monthly high-dose vitamin D supplementation was not found to prevent cardiovascular disease [27]. Also, in a randomized controlled study of 25,871 participants in the United States, with a median follow-up of 5.3 years, the incidence of cardiovascular events was not reduced after supplementation with vitamin D [28]. However, in another randomized controlled study of 90 patients with stable coronary artery disease residing in Beijing, the result indicated its benefit for coronary artery disease, suggesting it could be an adjunctive therapy for patients with coronary artery disease [18]. Participants with stable cardiovascular disease (n = 946) in San Francisco, with a median follow-up period of 8.0 years, low levels of 25-hydroxyvitamin D were found to be associated with cardiovascular events, but after adjustment, there was no independent association [29]. In subjects with heart failure, a case-control study with a case group (n = 3009) versus a control group (n = 46,825) [30] and an RCT study also showed contrasting results [31]. The design of the clinical study, the control of covariates, and the lack of follow-up time may limit the finding of actual results. Low and high levels of 25(OH)D in the general population (n = 247,574) in Copenhagen were associated with mortality in a nonlinear manner [15]. Another cohort study included from the UK Biobank study illustrated that elevated serum 25(OH)D levels in patients with CVD (n = 37,079) were independently associated with reduced mortality and nonlinearly in all-cause mortality [32]. A L-shaped relationship was obtained in a non-linear Mendelian randomization analyses of 44,519 CVD cases [33]. These were similar to our findings.

Approximately 25-57% of the US population has been investigated to be deficient in vitamin D [34]. Vitamin D deficiency is relevant to unfavorable bone outcomes [35]. The primary source of vitamin D in humans is that it is obtained in small amounts, primarily through skin synthesis under the influence of sunlight (i.e., UV) [36]. Others comes from fish and mushrooms [37]. Vitamin D deficiency may be associated with many risk factors include inflammatory pathways, nitric oxide regulation, oxidative stress, and renin-angiotensin-aldosterone system activation [37]. Vitamin D significantly reduces endothelial dysfunction and damage induced by oxidative stress [38]. 25(OH)D3 also regulates transcriptional endothelial NO synthase [39]. Vitamin D deficiency is also significantly associated with oxidative stress, inflammation, and major chronic diseases associated with aging [40], with impaired signaling causing hypertension [41]. It also has anti-inflammatory and anti-foam cell effects [42]. Some studies have illustrated that vitamin D supplementation may be beneficial in preventing and treating CAD in a porcine model [27]. Vitamin D deficiency-mediated signaling can overstimulate cardiomyocytes, including increased contractility and myocardial hypertrophy, and produce heart failure [43]. Vitamin D may also protect against stroke through receptor-mediated signaling pathways [44]. Based on the stratified analysis, we observed the advantage to reduce risk of all-cause mortality with the increase of serum 25(OH)D after natural log-transformed (4.31) in patients with prior cardiovascular disease, particularly in elderly patients (≥ 60 years old) race White BMI (25–30 kg/m2) hypercholesterolemia (yes) glycohemoglobin (< 7%) and family income-poverty ratio (1–3). Therefore, for patients with prior cardiovascular disease, we should consider the serum vitamin D level, particularly in a elderly patients (> 60 years old), White people, glycohemoglobin (< 7%), hypercholesterolemia and normal figure patients.

A recent paper reported no essential health benefits of vitamin supplementation in an elderly population [45]. It seems to suggest that vitamin D needs are not consistent among different populations.

In populations without vitamin D deficiency, a recent review summarized the results of Mendelian Randomization studies of vitamin D levels conducted during 2017–2020. In vitamin D-enriched adults, vitamin D supplementation alone did not provide health benefits, but vitamin D deficiency should permanently be corrected [46]. Another recent review showed that the level of biomarkers related to chronic heart metabolic diseases and cancer is significantly reduced by vitamin D [47]. In a survey of different countries, different diseases, and different supplements, it was found that current research disagrees with the recommendation of fish oil and vitamin supplementation to weaken the risk of disease in people without nutritional deficiencies [48] and that excessive vitamin D activity can also induce vascular calcification [49]. Our study also found that the protective effect decreased from strong to weak with increasing serum vitamin D concentration, reaching a maximum protective impact at a particular value. Compared with vitamin D after natural log-transformed (4.31–4.5), in patients without vitamin D deficiency, continued increases in vitamin D did not continue to increase the protective effect, but when severe vitamin D deficiency occurred, the risk of all-cause mortality is significant, a finding that provides evidence for a threshold value of serum vitamin D in patients with prior CVD. Vitamin D could be a cheap and safe adjunctive therapy due to broad safety profile [35].

Our study has some strengths. It is the first study to find a significant relationship between serum vitamin D levels and the risk of all-cause mortality in people with prior CVD using the US sample pool (NHANES 2007–2018). Our study had a large sample size to provide a better overview of the US population, adjusted for more confounding factors and laboratory indicators, and performed subgroup analyses and interactions to assess the true association between serum vitamin D and the risk of all-cause mortality in people with prior cardiovascular disease.

However, this study has some shortcomings. First, demographic data in the database with self-reported dietary data with questionnaire data might introduce bias and be unavoidable. Therefore, this sample only applies to populations with adults with prior cardiovascular disease. Second, other covariates not included may influence the results. Third, the relationship between serum vitamin D and different types of death was not explored in previous cardiovascular patients. In addition, not differentiating the specific type of cardiovascular event for which the patient was included in the study and its relationship with the end point evaluated. Finally, we only analyzed a single measurement of serum vitamin D levels, and concentrations of serum 25(OH)D in the state of health prior to the cardiovascular event remain unknown.

## Conclusions

After multivariable adjustment, increases in serum 25(OH)D levels in American adults with the prior cardiovascular disease show a L-shaped relationship with risk of all-cause mortality, and increases in serum 25(OH)D levels above a certain threshold do not continue to reduce risk of all-cause mortality. The causal relationship between serum levels of 25(OH)D and all-cause mortality in adults with cardiovascular disease needs further investigation.

## Electronic supplementary material

Below is the link to the electronic supplementary material.

Additional file 1: Appendix 1. Suggested MET Scores

## Data Availability

The datasets were available from NHANES 2007–2018 (https://www.cdc.gov/nchs/nhanes/ index. htm). The datasets are available from the corresponding author on reasonable request.

## Abbreviations

US: The United States
CVD: Cardiovascular disease
RAAS: The renin-angiotensin-aldosterone system
25OHD: 25-hydroxyvitamin D
NHANES: The National Health and Nutrition Examination Survey
NCHS: The National Center for Health Statistics
HPLC-MS/MS: High-performance liquid chromatography-tandem mass spectrometry
BMI: Body mass index
WBC: White blood cell
HDL: High-density lipoprotein
LDL: Low-density lipoprotein
AST: Aspartate aminotransferase
ALT: Alanine aminotransferase
SD: Standard deviations
HRs: Hazard ratios
Cis: Confidence intervals
PTH: Parathyroid hormone
RCT: Randomized controlled trial
