# Supplementary material for: Association between serum 25(OH)D and risk of all-cause mortality in adults with prior cardiovascular disease: a cohort study from NHANES 2007–2018
Source: BMC Cardiovasc Disord. 2023 May 6;23:240. doi: 10.1186/s12872-023-03257-0 (PMC10164325; doi:10.1186/s12872-023-03257-0)
Supplement: Supplementary file 1 — Additional file 1: Appendix 1. Suggested MET Scores [file 12872_2023_3257_MOESM1_ESM.pdf]

Additional file 1: Appendix 1. Suggested MET Scores.

**Variable**	**Label**	**Score**
PAD615	Vigorous work-related activity	8.0
PAD630	Moderate work-related activity	4.0
PAD645	Walking or bicycling for transportation	4.0
PAD660	Vigorous leisure-time physical activity	8.0
PAD675	Moderate leisure-time physical activity	4.0
